# Heat stroke

**DOI:** 10.1186/s40560-018-0298-4

**Published:** 2018-05-22

**Authors:** Toru Hifumi, Yutaka Kondo, Keiki Shimizu, Yasufumi Miyake

**Affiliations:** 1 Emergency Medical Center, Kagawa University Hospital, 1750-1 Ikenobe, Miki, Kita, Kagawa 761-0793 Japan; 2 Department of Emergency and Critical Care Medicine, Juntendo University, Urayasu Hospital, 2-1-1 Tomioka,Urayasu-shi, Chiba, 279-0021 Japan; 3 Emergency and Critical Care Center, Tokyo Metropolitan Tama Medical Centre, 2-8-29 Musashidai, Fuchu-shi, Tokyo, 183-8524 Japan; 4 Department of Emergency Medicine, Teikyo University School of Medicine, 2-11-1 Kaga, Itabashi-Ku, Tokyo, 173-8606 Japan; 5 Department of Emergency and Critical Care Medicine, St. Luke’s International Hospital, 9-1 Akashi-cho, Chuo-ku, Tokyo, 104-8560 Japan

**Keywords:** Heat stroke, Blood purification therapy, Anticoagulation, JAAM criteria, Core body temperature, Intravascular cooling

## Abstract

**Background:**

Heat stroke is a life-threatening injury requiring neurocritical care; however, heat stroke has not been completely examined due to several possible reasons, such as no universally accepted definition or classification, and the occurrence of heat wave victims every few years. Thus, in this review, we elucidate the definition/classification, pathophysiology, and prognostic factors related to heat stroke and also summarize the results of current studies regarding the management of heat stroke, including the use of intravascular balloon catheter system, blood purification therapy, continuous electroencephalogram monitoring, and anticoagulation therapy.

**Main body:**

Two systems for the definition/classification of heat stroke are available, namely Bouchama’s definition and the Japanese Association for Acute Medicine criteria. According to the detailed analysis of risk factors, prevention strategies for heat stroke, such as air conditioner use, are important. Moreover, hematological, cardiovascular, neurological, and renal dysfunctions on admission are associated with high mortality, which thus represent the potential targets for intensive and specific therapies for patients with heat stroke. No prospective, comparable study has confirmed the efficacy of intravascular cooling devices, anticoagulation, or blood purification in heat stroke.

**Conclusion:**

The effectiveness of cooling devices, drugs, and therapies in heat stroke remains inconclusive. Further large studies are required to continue to evaluate these treatment strategies.

## Background

Heat stroke is a life-threatening injury requiring neurocritical care, and there have been at least 3332 deaths attributed to heat stroke from 2006 to 2010 in the USA [1]. Regarding heat stroke, 28-day and 2-year mortality rates have been reported to be 58 and 71%, respectively [2]. In addition, the number of deaths from heat stroke has been reported to increase due to climate change [1]. By the 2050s, heat stroke-related deaths are expected to rise by nearly 2.5 times the current annual baseline of approximately 2000 deaths [2].

Unfortunately, heat stroke has not been comprehensively examined due to several possible reasons. First, while sepsis, acute respiratory distress syndrome (ARDS), and acute kidney injury (AKI) include simple and commonly used definitions, no universally accepted definition of heat stroke exist in the clinical settings. Second, because a large number of heat stroke victims are uncommon in the USA or European countries (ex. 1995, and 1999 in Chicago, 2003 in Paris) [2–5], clinical research has not been continuously conducted in these regions.

Several review articles regarding heat stroke focusing on critical care have been published in the early 2000s [6, 7]; moreover, additional new devise for cooling, blood purification therapy for renal/hepatic failure, continuous electroencephalogram (cEEG) monitoring, and the use of drugs, such as anticoagulants, for treating heat stroke have become readily available, and substantive clinical research regarding such devises/drugs has been published in the 2010s [8–13].

Thus, in the current review, we elucidate the definition/classification, pathophysiology, and prognostic factors associated with heat stroke and also summarize the results of current studies regarding the management of heat stroke, including the use of intravascular balloon catheter systems, blood purification therapy, cEEG monitoring, and anticoagulants.

## Review

### Definition and classification of heat stroke

Historically, heat stroke has been classified into two groups according to the presence or absence of exertion. Exertional heat stroke develops in able-bodied individuals, such as athletes, soldiers, or laborers, and performing rigorous physical activities [1]. In contrast, nonexertional heat stroke can develop during low-level physical activities among elderly, ambulatory individuals with comorbidities including obesity, diabetes, hypertension, heart disease, renal disease, dementia, and alcoholism [1].

To date, no universally accepted definition of heat stroke exists. The most commonly used definition of heat stroke worldwide is the Bouchama’s definition [6]. Bouchama has defined heat stroke as a core body temperature that rises above 40 °C, accompanied by hot dry skin and central nervous system abnormalities, such as delirium, convulsions, or coma. Heat stroke results from exposure to a high environmental temperature or from strenuous exercise [6]. Bouchama has also proposed an alternative definition of heat stroke on the basis of its pathophysiology, stating that heat stroke is a form of hyperthermia associated with a systemic inflammatory response that leads to a syndrome of multiorgan dysfunction, predominantly encephalopathy [6].

Pease et al. have reported an unusual heat wave that lasted 9 days in France in 2003 [14] and referred to the following criteria according to the Bouchama’s definition: the alteration of mental status (coma, delirium, disorientation, or seizures); a body core temperature of > 40.6 °C or a documented evidence of cooling before the first record temperature; a reliable history of compatible environmental exposure; and the presence of hot, dry, or flushed skin. In another study, Misset et al. defined heat stroke as “the presence of hyperthermia of >40.5°C” [15], but the phrase “core body temperature” was not included in their definition. Consequently, specific body temperature and the use of phrase “core body temperature” vary across studies.

**Fig. 1:**
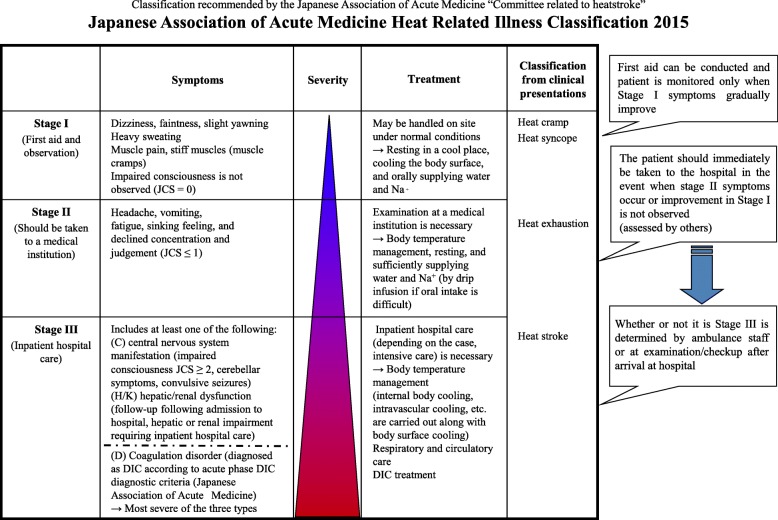
Japanese Association of Acute Medicine Heat-Related Illness criteria. DIC, disseminated intravascular coagulation; JCS, Japan Coma Scale

In Japan, the Japanese Association for Acute Medicine (JAAM) has collected data through a nationwide heat-related illness registry of patients diagnosed as having heat-related illnesses (including heat stroke) regardless of the core body temperature since 2006 [16, 17]. The JAAM has established and published the criteria for heat-related illnesses, including heat stroke, in 2014 [18] (Fig. 1).

Heat stroke was defined as patients exposed to high environmental temperature who met one or more of the following criteria:

I. Central nervous system manifestation (impaired consciousness with a Japan Coma Scale score of ≥ 2 [19], cerebellar symptoms, convulsions, or seizures);
II. Hepatic/renal dysfunction (follow-up following admission to hospital, hepatic or renal impairment requiring inpatient hospital care);
III. Coagulation disorder [diagnosed as disseminated intravascular coagulation (DIC) by the JAAM] [20, 21].

Apparently, the body temperature was not included in these diagnostic criteria because of several fatal cases of patients whose body temperatures were below 40 °C that were observed in clinical practice [22].

In 2016, the JAAM Heat Stroke (JAAM-HS) Committee launched a working group (JAAM-HS-WG) to analyze the collected megadata regarding heat-related illnesses. The JAAM-HS-WG further simplified the heat stroke classification [22]. The modified JAAM heat stroke definition included patients exposed to high environmental temperature and meeting at least one of the following criteria:

I. Glasgow Coma Scale (GCS) score of ≤ 14,
II. Creatinine or total bilirubin levels of ≥ 1.2 mg/dL,
III. JAAM DIC score of ≥ 4.

The difference between the definitions/classifications of heat stroke among Bouchama’s definition and the JAAM and JAAM-HS-WG criteria is summarized in Table 1.

**Table 1:** Comparison of Bouchama’s definition and the JAAM criteria for heat stroke

		Bouchama’s definition	JAAM criteria	JAAM-HS-WG criteria
Environment		Exposure to environmental heat (classic heat stroke)	Exposure to high environmental temperature	
Body temperature		Core body temperature > 40 °C	**–**	**–**
Organ dysfunction	Central nervous system	Delirium, convulsions, or coma	Impaired consciousness JCS ≥ 2, cerebellar symptoms, convulsive seizures	GCS score ≤ 14
	Coagulation	**–**	Diagnosed as DIC by JAAM	JAAM DIC score ≥ 4
	Liver	**–**	Follow-up after admission to hospital, hepatic or renal impairments requiring inpatient hospital care	Creatinine or total bilirubin levels ≥ 1.2 mg/dL
	Renal	**–**		
	Cardiovascular	**–**	**–**	**–**
	Respiratory	**–**	**–**	**–**

*GCS* Glasgow Coma Scale, *JAAM* Japanese Association of Acute Medicine, *JAAM-HS-WG* Japanese Association of Acute Medicine heat stroke committee working group, *JCS* Japan Coma Scale, *DIC* disseminated intravascular coagulation

## Pathogenesis

### Thermoregulation

A normal body temperature is maintained at approximately 37 °C by the anterior hypothalamus through the process of thermoregulation [23, 24]. Several mechanisms related to sweating, such as vaporization, radiation, convection, and conduction, function to cool the body surface [25]. As the body temperature increases, active sympathetic cutaneous vasodilation increases blood flow in the skin and initiates thermal sweating [26, 27]. Cutaneous vasodilation causes a relative reduction in intravascular volume, leading to heat syncope [28]. The loss of salts and water through sweat induces dehydration and salt depletion, which are associated with heat exhaustion and cramps unless appropriate supplementations of water and salt are initiated [28]. Further loss of salt and water impairs thermoregulation followed by the reduction of visceral perfusion due to shunt from the central circulation to the skin and muscles, resulting in organ failure [6, 28, 29]. Therefore, heat stroke is a condition of multiple organ failure caused by hot environment.

### Heat shock response

Heat shock proteins (HSP) are a family of proteins produced by nearly all cells in response to stressful conditions, including heat shock as well as other stresses, such as exposure to cold and ultraviolet light [6, 30]. Increased levels of HSPs, such as HSP70, are necessary for acquired heat tolerance. Moreover, the overexpression of HSP70 in response to heat stress can protect against organ dysfunction and reduces mortality in rats [30].

## Pathophysiology

Hyperthermia due to passive heat exposure facilitates the leakage of endotoxin from the intestinal mucosa to the systemic circulation as well as the movement of interleukin (IL)-1 or IL-6 proteins from the muscles to the systemic circulation [31]. This causes an excess activation of leukocytes and endothelial cells manifested by the release of various cytokines and high-mobility group box 1 protein (HMGB1), which is a prototypic alarmin (endogenous molecules that signal tissue and cellular damage). Together, these processes cause the systemic inflammatory response syndrome [6, 32, 33].

The inflammatory and coagulation responses to heat stroke, together with direct cytotoxic effects of heat, injure the vascular endothelium, causing microthromboses [6]. Platelet counts decrease because of microthrombosis, the secondary consumption of platelets, and hyperthermia-induced platelet aggregation. Heat stroke also suppresses platelet release from bone marrow due to megakaryocyte susceptibility to high temperature exposures. Heat stroke-induced coagulation activation and fibrin formation clinically manifest DIC.

## Prognostic factors

As mentioned above, because the definition of heat stroke varies across studies, detailed examinations, rather than mere results, are required to understand these study findings (Table 2).

**Table 2:** Prognostic factors

Study	Country	Patients	Number of patients	Outcomes	Factors
Hausfater et al. [34]	France	Nonexertional heatstroke (core body temperature > 38.5 °C)	1456	1-year mortality	Previous treatment with diuretics, living in an institution, age > 80 years, presence of cardiac disease or cancer, core body temperature > 40 °C, SBP < 100 mmHg, GCS score < 12, and transportation to hospital in ambulance
Argaud et al. [2]	France	Nonexertional heatstroke (core body temperature > 40 °C)	83	2-year mortality	Living at an institution, the use of long-term antihypertensive medication, presence of anuria, coma, or cardiovascular failure at admission
Misset et al. [15]	France	Heat stroke in Bouchama’s definition	345	Hospital death	At home or in a healthcare facility (vs. in a public location), high SAPS II score, initial high body temperature, prolonged prothrombin time and the use of vasoactive drugs within the first day in ICU, and patient management in an ICU without air conditioning
Tsuruta et al. [16]	Japan	Mechanically ventilated heat-related illness in JAAM-HS criteria	77	Poor outcome (death and incidence of sequelae)	SBP, SpO2 at scene, and arterial base excess
Hifumi et al. [22]	Japan	Heat stroke in JAAM-HS-WG criteria	705	Hospital death	SBP, GCS score, serum creatinine levels, and presence of DIC

*JAAM-HS-WG* Japanese Association of Acute Medicine heat stroke committee working group, *SBP* systolic blood pressure, *GCS* Glasgow Coma Scale, *SAPS II* Simplified Acute Physiology Score II score, *DIC* disseminated intravascular coagulation, *ICU* intensive care unit

Patients exposed to the August 2003 heat wave in Paris were examined to identify the prognostic factors, and several studies examining different populations have been published. Hausfater et al. have examined all patients who developed the core temperatures of > 38.5 °C, who were admitted to one of the emergency departments during the August 2003 heat wave in Paris. Previous treatment with diuretics, living in an institution, age > 80 years, the presence of cardiac disease or cancer, core temperature > 40 °C, systolic arterial pressure < 100 mmHg, GSC scale < 12, and transportation to hospital in ambulance were identified as prognostic factors associated with death for nonexertional heatstroke [34]. Argaud et al. examined long-term outcome in 83 patients with nonexertional heatstroke resulting from the August 2003 heat wave in Paris and having core temperatures > 40 °C. Multivariate cox proportional hazard model analysis revealed an independent contribution to 2-year mortality if patients were staying at an institution (hazard ratio (HR), 1.98; 95% confidence interval (CI), 1.05–3.71), if they used long-term antihypertensive medications (HR, 2.17; 95% CI, 1.17–4.05), or if they presented with anuria (HR, 5.24; 95% CI, 2.29–12.03), coma (HR, 2.95; 95% CI, 1.26–6.91), or cardiovascular failure (HR, 2.43; 95%CI, 1.14–5.17) at admission [2]. Misset et al. have conducted a questionnaire survey and a multivariate analysis, wherein the occurrence of heatstroke at home or in a healthcare facility (vs. in a public area), high Simplified Acute Physiology Score (SAPS) II score [35], initial high body temperature, prolonged prothrombin time, the use of vasoactive drugs within the first day in intensive care unit (ICU), and patient management in an ICU without air conditioning were independently associated with an increased risk of hospital death [15].

Tsuruta et al. have examined 77 mechanically ventilated patients with heat-related illnesses who met the JAAM-HS criteria. Their systolic blood pressure (SBP) and SpO2 at scene and arterial base excess were identified as independent risk factors for poor outcomes (death and with sequelae).

Hifumi et al. have examined 705 patients who met the JAAM-HS-WG criteria for heat stroke and observed that the hospital mortality was 7.1% (50 patients) [22]. Multiple regression analysis revealed that hospital mortality was significantly associated with SBP (odds ratio (OR), 0.99; 95% CI, 0.98–0.99; *p* = 0.026), GCS score (OR, 0.77; 95% CI, 0.69–0.86; *p* < 0.01), serum creatinine levels (OR, 1.28; 95% CI, 1.02–1.61; *p* = 0.032), and the presence of DIC on admission (OR, 2.16; 95% CI, 1.09–4.27; *p* = 0.028) [22].

According to the detailed analysis of risk factors, careful attention should be paid for the prevention of heat stroke in patients living in a healthcare facility, aged > 80 years, and previously treated with diuretics. Moreover, because hematological, cardiovascular, neurological, and renal dysfunctions on admission are associated with high mortality, these dysfunctions represent potential targets for intensive and specific therapies for patients with heat stroke.

## Treatment

Heat stroke progresses to multiorgan dysfunction syndrome; therefore, rapid, effective cooling followed by close monitoring and specific treatment for injured organs are fundamental to treatment success.

### Initial cooling

#### Target temperature of initial cooling

There is no evidence to support a specific temperature end point; however, a rectal temperature of 39.4 °C has been used in large series and has been proven to be safe [6].

#### Initial cooling method

To date, several cooling methods are available in the clinical settings, including immersion [36], evaporation [37], and the use of cold water bladders, gastric and rectal lavage [38], and noninvasive cooling systems [39]. However, there is no evidence supporting the superiority of any one cooling method for patients with heat stroke [6]. An intravascular balloon catheter system has been approved in the USA for therapeutic core cooling and rewarming in humans during or following cardiac or neurological surgery and following stroke [40]. However, a few cases have reported the use of intravascular cooling for heat stroke [41, 42]. Hamaya et al. have reported for the first time a good recovery in a case of severe heat stroke, followed by multiple organ dysfunction, which was successfully treated through initial intravascular cooling [12]. In this case, at an average rate of 0.1 °C/min, the core temperature of the patient’s body reached 38.8 °C after just 17 min. Yokobori et al. have conducted a prospective study examining the feasibility and safety of a convection-based intravascular cooling device (IVC) in patients with severe heat stroke. Comparison between IVC plus conventional cooling (CC) and CC was made in patients with severe heat stroke. The IVC group showed a significant decrease in the Sequential Organ Failure Assessment score during the first 24 h (from 5.0 to 2.0, *P* = 0.02). Moreover, all patients in the IVC group (*N* = 9) experienced favorable outcomes defined as modified Rankin scale score of 0–2 at discharge and at 30 days after the admission. Their findings indicate that accurate temperature management may prevent organ failure and produce better neurological outcomes. The Fukuoka University Hospital group has used extracorporeal circulation with hemodiafiltration circuits for cooling patients with severe heat stroke and has reported improved cooling efficiency [43]. To date, there have been no prospective, comparative studies confirming the superiority of the initial cooling method. Intravascular balloon catheter system does not result in cutaneous vasoconstriction as external cooling does, but it requires the placement of cooling balloon.

### Management for organ dysfunctions in ICU

#### Central nervous system dysfunction

Nakamura et al. have examined central nervous system sequelae of heat-related illnesses and have observed that 22 of 1441 cases (1.5%) exhibited the central nervous system sequelae of heat-related illnesses. Heatstroke patients presenting with lower GCS scores and higher body temperatures at admission were more likely to experience central nervous system sequelae and required longer cooling times to achieve the target body temperature. Therefore, rapid cooling followed by neuromonitoring might be associated with the neurological sequelae of heat stroke.

Recently, Hachiya et al. have reported the usefulness of cEEG in patients with severe heat stroke complicated with multiorgan failure [13]. The patients developed a persistent disturbance of consciousness; therefore, cEEG monitoring was applied. cEEG monitoring confirmed triphasic waves, which indicated hepatic failure as the cause of the persistent disturbance of consciousness. The patient’s condition improved following an artificial liver support therapy [13]. Thus, no prospective, comparable study has revealed the adequate neuromonitoring and the effect of temperature control on central nervous system.

#### Coagulation disorder

##### Anticoagulation therapy

- Antithrombin: Pachlaner et al. have reported good recovery in a patient with near-fatal heat stroke treated with type III antithrombin (AT-III) [44]. On admission, although the patient’s AT-III activity was 98%, a treatment with AT-III concentrate was initiated within 24 h due to DIC, which was aimed toward achieving supranormal plasma concentrations. Plasma AT concentrations were maintained at > 120% by continuous intravenous supplementation [44]. Additionally, in a rat model of heat stroke, AT-III treatment decreased serum cytokines (IL-1 β, tumor necrosis factor-α, and IL-6), and HMGB1 [45]. Thus, prospective studies will be needed to confirm the efficacy of AT-III supplementation in improving the clinical outcome of heat stroke.
- Thrombomodulin (TM): Recombinant soluble thrombomodulin α (rTM), which is currently under phase III clinical trials for use in patients with severe sepsis, could also be a candidate for the treatment of heat stroke-induced DIC [46] because it serves as a negative feedback regulator of blood coagulation [47]. In basic research, rTM prevents heat stroke by inhibiting HMGB1 [48]. Sakurai et al. have reported (in Japanese) two cases of good recovery from heatstroke-induced DIC, which were successfully treated with TM administration [49]. Prospective studies will be needed to confirm the efficacy of rTM.

#### Hepatic/renal dysfunction

##### Blood purification therapy

Blood purification therapy has not been discussed in the two previously reported review articles; however, good recovery cases have been reported in Japan [6, 7].

Ikeda et al. have reported three cases of survival following multiorgan failure secondary to heat stroke that was treated with blood purification therapy, including continuous venovenous hemofiltration and plasma exchange (PE) [8]. Blood purification therapy removes proinflammatory cytokines related to heat stroke [8]. Chen et al. have conducted a retrospective study including 33 patients with severe exertional heat stroke and have compared clinical effects of continuous renal replacement therapy (CRRT) and routine therapy in these patients. They reported significantly lower 30-day mortality in the CRRT group than in the control group (15.2% vs.45.5%, *p* = 0.029) although initial APACHE II scores in both groups were similar [10].

Recently, Inoue et al. have reported a case of severe exertional heat stroke with multiple organ failure that was successfully treated with continuous plasma diafiltration (PDF) [11]. PDF is a blood purification therapy in which PE is performed using a selective membrane plasma separator while the dialysate flows outside the hollow fibers. This separator has a small pore size (0.01 mm) and a sieving coefficient of 0.3 for albumin, which can selectively remove low- or intermediate-molecular weight albumin-bound substances [50–52].

In the clinical practice, decisions to continue blood purification therapy are difficult because this therapy is time-consuming and costly. Yonemitsu et al. have published a case report and literature review of cases of heat stroke treated with blood purification therapy [53]. The review includes several survival cases treated more than three times with PE; therefore, withdraw therapy following only a few trials. No prospective, comparable study has confirmed the efficacy of blood purification in heat stroke.

#### Cardiovascular dysfunction

Hart et al. have observed that supplementary vasoactive agents necessary to elevate blood pressure were associated with both high mortality rates and neurologic disability in patients with heat stroke [54]. Misset et al. have demonstrated that the use of vasoactive drugs within the first 24 h of admission to ICU was an independent factor associated with mortality. These findings suggest a close association between hypotension and poor outcomes. To date, no prospective, comparable study has confirmed the efficacy of targeted fluid administration or specific vasoactive drugs in heat stroke.

## Prevention

It would be acceptable to consider prevention, rather than the treatment of organ dysfunctions, because therapeutic options for organ dysfunction are rather limited even in the late 2010s, as described above. Nonetheless, heat-related deaths and illnesses are preventable [6, 55]. Heat stroke prevention strategies, such as using air conditioner; limiting outdoor activities during the daytime; consuming ample fluids; wearing loose-fitting light-colored clothing, being aware of medication side effects that may cause fluid losses, decrease sweating, or decreased heart rate; and never leaving impaired adults or children in a car unattended, are important [55]. Centers for Disease Control and Prevention has uploaded a video titled “How to Stay Cool in Extreme Heat” to YouTube [56].

## Conclusions

In the present review, we elucidated the clinical diagnosis of heat stroke. Regarding the definition/classification of heat stroke, the Bouchama’s definition and the JAAM criteria are the two available systems. Intravascular cooling devises provided rapid cooling in the small number of heat stroke patients. Although few case reports and retrospective case-series for the use of anticoagulation and blood purification therapies have been reported, particularly in Japan, no prospective, comparative study has been conducted to date. Further large studies are warranted to evaluate these treatment strategies among patients with heat stroke.

## Abbreviations

AKI: Acute Kidney Injury
ARDS: Acute Respiratory Distress Syndrome
CC: Conventional cooling
cEEG: Continuous Electroencephalogram
CRRT: Continuous Renal Replacement Therapy
DIC: Disseminated Intravascular Coagulation
GCS: Glasgow Coma Scale
HMGB1: High-mobility group box 1
HSP: Heat shock proteins
ICU: Intensive care unit
IVC: Convection-based intravascular cooling device
JAAM: Japanese Association for Acute Medicine
JAAM-HS-WG: Japanese Association of Acute Medicine heat stroke committee working group
JCS: Japan Coma Scale
PDF: Plasma diafiltration
PE: Plasma exchange
SAPS II: Simplified Acute Physiology Score II score
SBP: Systolic blood pressure
